# Comparing eating and mealtime experiences in families of children with autism, attention deficit hyperactivity disorder and dual diagnosis

**DOI:** 10.1177/13623613241277605

**Published:** 2024-09-12

**Authors:** Zoe Matthews, Donna Pigden-Bennett, Teresa Tavassoli, Sarah Snuggs

**Affiliations:** University of Reading, UK

**Keywords:** ADHD, autism spectrum disorders, family functioning and support, school-age children

## Abstract

Shared family meals are associated with family functioning and thought to protect against disordered eating in families with neurotypical children. Limited research, however, has examined the mealtime structure or experience in families with children with autism (Autism spectrum condition) and/or attention deficit hyperactivity disorder, despite eating behaviour differences in these populations. This study sought to compare children’s eating behaviours and caregivers’ mealtime experiences between families with neurotypical children and those with Autism spectrum condition, attention deficit hyperactivity disorder and dual diagnosis (Autism spectrum condition + attention deficit hyperactivity disorder). Think-Aloud methods established validity for the Children’s Eating Behaviour Questionnaire, Meals in our Household and Perceived Stress Scale measures (*N* = 9), prior to administering these to caregivers of children aged 3–15 (*N* = 351). Neurodevelopmental condition groups (Autism spectrum condition, *n* = 80; Attention deficit hyperactivity disorder, *n* = 88; Autism spectrum condition + attention deficit hyperactivity disorder, *n* = 65) reported higher food fussiness, emotional undereating, problematic child mealtime behaviours, dietary concerns, caregiver and spousal stress, and less conventionally structured mealtimes than neurotypical families (*n* = 118). Attention deficit hyperactivity disorder and Autism spectrum condition + attention deficit hyperactivity disorder groups reported higher food responsiveness, problematic behaviour and caregiver stress than the Autism spectrum condition group. Conversely, Autism spectrum condition and Autism spectrum condition + attention deficit hyperactivity disorder groups reported lower food enjoyment and mealtime structure than the attention deficit hyperactivity disorder group. Distinct eating and mealtime patterns in neurodevelopmental condition families may adversely impact family functioning, emphasising the need for bio-psychosocial approaches and transdiagnostic mealtime support.

**Lay abstract:**

Children with neurodevelopmental conditions like autism and attention deficit hyperactivity disorder may experience eating difficulties and related health issues later in life. Sharing family meals can help prevent these issues developing, but most studies have looked at families with neurotypical children. Our goal was to learn more about how families of children with autism, attention deficit hyperactivity disorder and both conditions (autism + attention deficit hyperactivity disorder) experience mealtimes. We developed an online survey asking caregivers about their child’s eating, mealtime experience and if they experienced stress. We tested it with nine caregivers and made improvements based on their feedback before recruiting 351 caregivers to complete the main survey. We found that families of children with neurodevelopmental conditions experienced greater food fussiness, emotional undereating, ‘problematic’ child mealtime behaviours, dietary concerns, higher stress for caregivers and spouses and less frequent conventionally structured mealtimes compared to those without these conditions. Families of children with attention deficit hyperactivity disorder and autism + attention deficit hyperactivity disorder reported greater appetite, ‘problematic’ mealtime behaviours and increased stress for caregivers and spouses compared to families of children with autism. Meanwhile, families of children with autism and autism + attention deficit hyperactivity disorder reported less enjoyment of food and less structured mealtimes compared to those with attention deficit hyperactivity disorder. Our findings highlight that families of children with neurodevelopmental conditions, particularly those with autism + attention deficit hyperactivity disorder, have different mealtime experiences and eating behaviours compared to those with neurotypical children. These families may benefit from support at mealtimes. Learning why people do or do not participate in shared family meals will be crucial to developing improved mealtime support in the future.

## Introduction

Autism spectrum condition^1^ (ASC) and attention deficit hyperactivity disorder (ADHD) are prevalent childhood neurodevelopmental conditions (NDC; Polanczyk et al., 2014), sharing significant heritability and co-occurrence (Lichtenstein et al., 2010). Between 59% and 83% of autistic children have an ADHD diagnosis, while 30%–60% of children with ADHD present with clinically significant autistic traits (Deserno et al., 2022; Joshi et al., 2020). ASC and ADHD have been associated with different strengths, difficulties and support needs (Bottema-Beutel et al., 2021). They also share some overlapping features (Miller et al., 2019; Stergiakouli et al., 2017). Children with dual ASC and ADHD diagnoses (ASC + ADHD) have been described as a ‘high need’ population (Hong et al., 2021; Zablotsky et al., 2020) and may experience greater social, adaptive and emotional functioning differences compared to ASC or ADHD diagnoses alone (Elwin et al., 2020; Sainsbury et al., 2022; Yerys et al., 2019). Hollingdale et al. (2020) propose that children with ASC + ADHD may combine the unique profiles associated with each condition, following the theory of additivity. However, limited research to date has focussed on ASC + ADHD specific needs (Antshel & Russo, 2019).

Children’s eating behaviour has been widely studied across the general population (Scaglioni et al., 2018; Taylor & Emmett, 2019; Wolstenholme et al., 2020a) and conceptualised into appetitive profiles of ‘food approach’ and ‘food avoidance’ traits (Wardle et al., 2001). Certain child eating behaviours correlate with specific health outcomes (Kininmonth et al., 2021). ‘Food approach’ traits such as emotional overeating, characterised by a greater appetite and interest in food, have been associated with child obesity (Kininmonth et al., 2021; Santos et al., 2011). ‘Food avoidant’ traits, such as food selectivity, are indicative of a smaller appetite and lower interest in food and may be predictive of avoidant/restrictive food intake disorder (ARFID), anorexia nervosa, bulimia nervosa and binge eating disorder in later life (Herle et al., 2020; Nicely et al., 2014; Nicholls & Viner, 2009). Children with ASC and ADHD may be at increased risk of developing eating disorders (Kaisari et al., 2017; Nickel et al., 2019; Ravi & Khan, 2020), however, this relationship is not yet sufficiently understood (Mayes & Zickgraf, 2019).

Recent research indicates children with ASC or ADHD may experience eating difficulties beyond typical developmental trajectories (Page et al., 2022; Smith et al., 2020), and may be less responsive to conventional interventions to support these (Sharp et al., 2013). Food selectivity in autistic children has consistently been reported (Rodrigues et al., 2023) and may be more persistent and frequent compared to neurotypical (NT) children (Bandini et al., 2017; Suarez et al., 2014; Wallace et al., 2018). A similar profile has been identified in children with ADHD traits (Harris et al., 2022; Thorsteinsdottir et al., 2021). Sensory reactivity differences, which are a diagnostic criterion for ASC, and common in ADHD, may underlie food selectivity (Cermak et al., 2010; Nimbley et al., 2022; Smith et al., 2020). For children with ASC + ADHD, the current literature is limited, however, in a twin study of 9- to 12-year-olds, Råstam et al. (2013) found that while eating difficulties were significantly higher for autistic children and those with ADHD compared to the general population, significantly more eating difficulties were reported for the ASC + ADHD group compared with ASC or ADHD alone.

Distinct eating behaviour phenotypes for ASC, ADHD and ASC + ADHD have been proposed (Fuemmeler et al., 2020; van’t Hof et al., 2020) but existing studies have tended to focus on narrow age ranges or behaviours (Thorsteinsdottir et al., 2021). Food avoidant traits have been associated with ASC populations (Baraskewich et al., 2021; Kral et al., 2015), while Nadeau et al. (2022) identified subgroups of selective eating (food avoidance) and selective overeating (food approach and avoidance) within autistic children. Food approach behaviours have consistently been associated with ADHD traits in younger children (Leventakou et al., 2016; Leventakou et al., 2022). Harris et al. (2022) conducted a prospective population-based study to examine these phenotypic differences, categorising children into subgroups based on caregiver-reported ASC and ADHD traits. The group with ‘high’ ADHD traits reported more food approach behaviours, while children with ‘high’ ASC traits demonstrated more food avoidant behaviours than the ‘low’ ASC and ADHD traits comparison group at ages 6 and 10. Children in the ‘high’ ASC + ADHD traits group expressed both more food avoidant and food approach behaviours than the comparison group, with some elevated levels compared to ASC or ADHD groups. However, these findings await replication in clinically diagnosed populations.

Understanding the role of the family mealtime environment affords further insight into how eating behaviours are shaped in childhood. Shared family meals practices are widely associated with decreased risk of eating disorders (Dallacker et al., 2019; Hamilton & Hamilton Wilson, 2009) and enhanced family functioning (Robson et al., 2020). Sharing family meals more frequently in childhood may affect eating behaviours and nutritional health in later life (Harrison et al., 2015; Scaglioni et al., 2018). However, a positive mealtime atmosphere has been identified as a key component associated with such positive outcomes (Dallacker et al., 2019). Conversely, a negative mealtime atmosphere may indicate a broader negative family climate, which may be a potential risk factor for childhood obesity (Turner et al., 2005). Families with autistic children have been found to eat fewer shared family meals and have more concerns around their child’s weight compared to families with NT children, although the relationship between these is yet to be explored (Tahech et al., 2024).

Further complexities have been revealed in the literature considering specific differences in eating and mealtime behaviours between autistic and NT children (Nadon et al., 2011b; Page et al., 2022). Parents of autistic children aged 3–11 years reported higher food selectivity associated with specific mealtime behaviours (e.g. overstuffs mouth, refuses to stay seated), concern about child’s diet, spousal stress and influence of the child’s food preferences on what other family members ate compared to families of NT children (Anderson et al., 2012; Curtin et al., 2015). Sustaining children’s mealtime participation to facilitate shared family meals may require considerable caregiver and familial intervention (Ausderau et al., 2019; Suarez et al., 2014). Caregiver stress in families with autistic children may be heightened through such mealtime experiences (Burkett et al., 2021; Lim et al., 2021), and indeed this is supported in qualitative research (Ausderau & Juarez, 2013; Fiese & Schwartz, 2008). However, some families of autistic children have reported experiencing resilience and joy in the face of adversity around family meals (Curtiss & Ebata, 2019).

Little is currently understood about mealtime characteristics in children with ADHD, but limited research indicates behavioural patterns that merit further investigation (Kaisari et al., 2017; Nazar et al., 2016). Research to date has focussed on the effect of diet on ADHD traits (Pelsser et al., 2017); however, a prospective population-based study suggested that the direction of association may also be reversed (Mian et al., 2019). Higher ADHD traits at age 6 predicted poorer diet at age 8, whereas diet quality at age 8 did not predict ADHD traits at age 10 when accounting for confounding variables. Differences in eating and mealtime patterns characterised by consumption of more irregular and frequent meals and sugary drinks, have also been identified in 6- to 10-year-old boys with ADHD compared to an age-matched NT group (Ptacek et al., 2014). Less is known about mealtime experiences in families of children with ASC + ADHD (Ciesielski et al., 2020) although due to shared phenomenology with ASC, some similarities may exist (Antshel & Russo, 2019; Wallace et al., 2021).

Differences in behavioural conduct and emotional regulation in children with ADHD (Graziano & Garcia, 2016) have been associated with higher daily parenting stress (Wiener et al., 2016). Mealtimes may factor into the difficulties that caregivers of children have reported in managing child behaviour at home (Leitch et al., 2019), yet no study to date has considered mealtime experiences, parental stresses and concerns in families of children with ADHD or ASC + ADHD. Understanding eating behaviours and the family mealtime environment is critical to supporting family functioning, wellbeing and health outcomes in neurodivergent populations (Ciesielski et al., 2020; Lim et al., 2021).

This study compares mealtime experiences in families of NT children to those with ASC, ADHD and ASC + ADHD aged 3–15 years. We hypothesise unique differences in child eating behaviours, mealtime environments and caregiver stresses and concerns between NT and NDC groups. Due to the exploratory nature of the study, direction is not specified. However, we anticipate more pronounced differences in the ASC + ADHD group compared to ASC and ADHD groups.

## Methods

This study was approved by the University of Reading Research Ethics Committee (SREC 2022-056-SS).

### Design

A cross-sectional online caregiver-report survey was designed to quantify mealtime characteristics of families of children with and without NDC. Preliminary work was undertaken to evaluate face and content validity using Think-Aloud methods (French et al., 2007). Think-Aloud is a cognitive interviewing method where participants verbalise their thoughts while completing tasks. Increasingly employed in health psychology research to evaluate usability, face and content validity of measures, it helps researchers understand cognitive processes, identify comprehension issues and reduce interviewer bias (Castle et al., 2021; Usher-Smith et al., 2021). Nine caregivers (seven females, two males; *M* age = 46.7 years; *SD* = 6.7) of children (six with NDC, three NT; *M* age = 10.5 years; *SD* = 3.1) completed a cognitive interview, each lasting approximately 45 minutes (see Supplementary Information for participant demographics). Results were analysed following Think-Aloud precepts (Aujla et al., 2020; Gardner et al., 2020). Minor survey adjustments were made to improve usability and accessibility, while leaving validated measures intact. Adjustments reflected alignment with UK context, pronoun sensitivities (e.g. ‘they’ instead of ‘him or her’) and re-ordering of words to facilitate comprehension (see Supplementary Information for questionnaire adjustments).

### Participants

A sample comprising 391 primary caregivers of children aged 3–15 with a primary clinical diagnosis of ASC, ADHD, ASC + ADHD or an NT child without any NDC diagnosis completed an online survey. Caregiver report of clinical diagnosis was considered sufficient for inclusion in respective ASC, ADHD and ASC + ADHD groups. Recognising diagnostic delay and under-reporting in children with ADHD (Young et al., 2021) and ASC (Parr et al., 2021), children reported to be on the diagnostic referral pathway and who reached the clinical cut-off threshold using screening tools considered appropriate for referral for specialist diagnostic assessment, were included in the respective clinical groups. For the Autism Spectrum Quotient (AQ-10) Child and Adolescent (Auyeung et al., 2008), one point was awarded for each reported characteristic up to a maximum score of 10 and cut-off point ⩾6 (Allison et al., 2012). For the Strengths and Weaknesses of ADHD-symptoms and Normal-behaviour (SWAN; Swanson et al., 2012), categorical scoring was adopted. A maximum score of 9 and cut-off threshold of ⩾6 indicated presence of ADHD-I (inattentive) and ADHD-HI (hyperactive/impulsive) features. A maximum of 18 with cut-off score of ⩾6 in both subscales indicated presence of ADHD-C (combined) features (Brites et al., 2015). Reaching the cut-off scores for both AQ-10 and SWAN indicated ASC + ADHD.

Participants were recruited from regional and national caregiver and NDC support groups, mainstream and specialist schools, social media, and snowball sampling. As 70%–90% of individuals with NDC may have one or more co-occurring psychiatric condition (Gillberg et al., 2004; Gnanavel et al., 2019; Pehlivanidis et al., 2020), children with secondary co-occurring diagnoses were included. Eleven participants who reported that their children were on the diagnostic pathway but did not meet clinical thresholds and 29 with missing critical data or implausible responses were excluded. A final sample of 351 participants with adequate group sizes provided sufficient power for the study. Caregiver and child demographics are reported in Tables 1 and 2 (see Supplementary Information for additional details).

**Table 1.** Caregiver demographics.

Demographics	ASC (*n* = 80)	ADHD (*n* = 88)	ASC + ADHD (*n* = 65)	NT (*n* = 118)	Total (*N* = 351)
Age range in years (%)					
16–20	1 (1.3)	0	0	0	1 (0.3)
20–29	4 (5.0)	6 (6.8)	4 (6.2)	4 (3.4)	18 (5.1)
30–39	28 (35.0)	41 (46.6)	26 (40.0)	41 (34.7)	136 (38.7)
40–49	37 (46.3)	33 (37.5)	24 (36.9)	59 (50.0)	153 (43.6)
50–59	10 (12.5)	7 (8.0)	10 (15.4)	14 (11.9)	41 (11.7)
60–69	0	1 (1.1)	1 (1.5)	0	2 (0.6)
Mean age (*SD*)^a^	40.9 (8.0)	39.5 (7.9)	41.1 (8.7)	41.5 (7.2)	40.8 (8.0)
Relationship to child (%)					
Mother	73 (91.3)	85 (96.6)	61 (93.8)	109 (92.4)	328 (93.4)
Father	6 (7.5)	2 (2.3)	2 (3.1)	8 (6.8)	18 (5.1)
Other	1 (1.3)	1 (1.1)	2 (3.1)	0	4 (1.2)
Ethnicity (%)					
White	73 (91.3)	83 (94.3)	65 (100)	110 (93.2)	331 (94.3)
Asian/Asian British	0	3 (3.4)	0	1 (0.8)	4 (1.1)
Black/Black British	0	1 (1.1)	0	1 (0.8)	2 (0.6)
Mixed/multiple ethnic groups	5 (6.3)	0	0	3 (2.5)	8 (2.3)
Other/Prefer not to say	2 (2.5)	1 (1.1)	0	3 (2.5)	6 (1.8)
Highest level of education completed (%)					
Secondary education	5 (6.3)	4 (4.5)	5 (7.7)	7 (5.9)	21(6.0)
Further education	32 (40.0)	26 (29.5)	18 (27.7)	23 (19.5)	99 (28.2)
Higher education	20 (25.0)	31 (35.2)	31 (47.7)	47 (39.8)	129 (36.8)
Postgraduate education	22 (27.5)	24 (27.3)	10 (15.4)	40 (33.9)	96 (27.4)
Prefer not to say	1 (1.3)	0	1 (1.5)	1 (0.8)	3 (0.9)

*Note.* Specific data on socioeconomic status were not recorded. ASC: children with autism; ADHD: attention deficit hyperactivity disorder; *SD*: standard deviation.

a Calculated using median scores of participants’ age ranges.

**Table 2.** Child demographics.

Demographics	ASC (*n* = 80)	ADHD (*n* = 88)	ASC + ADHD (*n* = 65)	NT (*n* = 118)	Total (*N* = 351)
Mean age (*SD*)	9.6 (3.2)	9.3 (3.1)	9.9 (2.8)	8.6 (3.9)	9.3 (2.4)
Gender (%)					
Male	56 (70.0)	72 (81.8)	56 (86.2)	66 (55.9)	250 (71.2)
Female	23 (28.7)	16 (18.2)	7 (10.8)	52 (44.1)	98 (27.9)
Non-binary	1 (1.3)	0	0	0	1 (0.3)
Other/prefer not to say	0	0	2 (3)	0	2 (0.6)
Ethnicity (%)					
White	74 (92.5)	78 (88.6)	63 (96.9)	106 (89.9)	321 (91.5)
Asian/Asian British	0	3 (3.4)	0	1 (0.8)	4 (1.1)
Black/Black British	1 (1.3)	1 (1.1)	0	0	2 (0.6)
Mixed/Multiple ethnic groups	3 (3.8)	4 (4.5)	1 (1.5)	9 (7.6)	17 (4.8)
Other	1 (1.3)	0	1 (1.5)	1 (0.8)	3 (0.9)
Prefer not to say	0	1 (1.1)	0	1 (0.8)	2 (0.6)

*Note.* ASC: children with autism; ADHD: attention deficit hyperactivity disorder; *SD*: standard deviation.

### Measures

In the absence of a universal measure of child eating and mealtime behaviours and environments in families (Mayes & Zickgraf, 2019), a cross-sectional caregiver-report survey was created incorporating three validated questionnaires.

#### The Children’s Eating Behaviour Questionnaire (CEBQ)

The CEBQ is a 35-item measure assessing ‘food approach’ and ‘food avoidant’ behaviours, with four subscales for food avoidance: satiety responsiveness (ability to regulate food intake), slowness in eating, food fussiness (food selectivity) and emotional undereating (eating less in response to heightened emotions; Wardle et al., 2001). Three subscales of interest assess food approach behaviours: food responsiveness (FR; readiness to eat), enjoyment of food (EF) and emotional overeating (EOE; eating more in response to heightened emotions). The ‘desire to drink’ scale was not deemed relevant to the research question. Higher Likert-type scale scores indicate higher levels of the behaviour. The CEBQ demonstrates good test/retest reliability (α = 0.78–0.92) and internal consistency across subscales (α = 0.74–0.91) in autistic, ADHD and NT populations (Aykutlu & Görker, 2019; Harris et al., 2022; Wardle et al., 2001).

#### The Meals In Our Household Questionnaire (MIOH)

The MIOH is a 50-item measure assessing the family mealtime environment and child behaviours (Anderson et al., 2012). It demonstrates acceptable internal consistency (α = 0.77; Anderson et al., 2012). Five of six subscales were of interest to this study: structure of family meals, for example, ‘Everyone in our household eats something different at meals’; problematic child mealtime behaviours, for example, ‘My child refuses to come when it is time to eat’ and ‘How much of a problem is it that your child refuses to come when it is time to eat?’ parents’ concern about child’s diet, for example, ‘My child is not getting good nutrition’; spousal stress related to child’s mealtime behaviour, for example, ‘My partner does not enjoy eating with my child’; and influence of child’s food preferences on what other family members eat, for example, ‘My child’s food preferences influence what I myself eat’. Higher Likert-type scale scores for structure of family meals (SFM) indicate increased frequency of exposure to conventionally structured family meals. For the remaining scales, higher scores indicate higher levels of behaviour, stress or concern. The Problematic Child Mealtime Behaviours (PMB) score combines two summed scores indicating frequency of the child’s behaviour *and* caregiver perception of how problematic they find that behaviour. An additional item of caregiver perception of stress was included based on Curtin and colleagues’ (2015) recommendation.

#### The Perceived Stress Scale-Short Form (PSS-4)

The PSS-4 is an established 4-item global measure of perceived stress (Cohen et al., 1983), with acceptable internal consistency (α = 0.74; Vallejo et al., 2018). Higher scores indicate greater perceived stress levels.

### Procedure

Participants accessed the anonymous survey, participant information and consent through JISC Online Surveys (https://www.onlinesurveys.ac.uk/) from May to June 2022. The survey, active for 1 month, took approximately 30 minutes to complete. The study debriefs advised participants to contact their GP for child-related concerns. Participants could enter a randomised draw for one of four £25 Amazon vouchers.

### Analysis

Cronbach’s Alpha was calculated to assess internal validity of outcome measures (CEBQ, MIOH, PSS-4). Group descriptive statistics were run and assumptions for analysis of variance (ANOVA) and analysis of covariance (ANCOVA) were assessed and satisfied for each dependent variable. A series of one-way ANOVA tests were subsequently conducted in IBM SPSS Statistics 28 to examine group differences in eating and mealtime behaviours. Bonferroni corrected alpha level (*p* < .007) was applied to the *F* statistic to account for multiple comparisons (Kim, 2015). Post hoc tests adjusted for multiple comparisons were reported (*p* < .05; two-tailed) where significant group differences were found. Due to unequal group sizes and Levene’s test indicating inhomogeneity of variances for some measures, Welch’s (1951) *F* and Games Howell post hoc tests were calculated to retain power, reducing Type I error risk (Rusticus & Lovato, 2014; Tomarken & Serlin, 1986). Partial eta squared (η^2^) effect sizes were reported (small effect = .001, medium = .06 and large = .14; Serdar et al., 2021). ANCOVA tests were subsequently run to examine whether significant effects remained after adjusting for the effect of age, which is known to influence appetitive traits and mealtime behaviours (Birch et al., 2007; Nadon et al., 2011a, 2011b).

### Community involvement

Both first authors have lived experience and/or close family members with ADHD or ASC. Neurodivergent adults and family members of children with NDC contributed to the study design and measures through the Think Aloud preliminary work. Community organisations supported participant recruitment, and participants could opt in to receive study results in lay article format.

## Results

### Assumptions

Outcome variables met the assumptions of linearity, independence of observations and absence of extreme outliers to proceed with parametric tests to examine differences between NDC and NT groups (Ghasemi & Zahediasl, 2012; Lee & Lee, 2018). Normality was assumed according to the Central Limit Theorum (Anderson, 2010). ANOVA has been found to be relatively robust to violations, maintaining good Type I error rates when studies are adequately powered (Harwell & Serlin, 1988; Owen & Froman, 1998; Schmider et al., 2010).

Groups showed no differences in child age, ethnicity, dietary restrictions, number of siblings, caregiver age, ethnicity, gender, household structure or education level (*p* > .05). More males than females were in the sample, χ^2^(3,347) = 28.18, *p* < .001, reflecting the prevalence of NDC diagnosis in boys (Baio et al., 2018; Faraone et al., 2015; Jang et al., 2013). The additional assumptions of independence and homogeneity of regression slopes were met to proceed with ANCOVA to ascertain whether the significant main effects remained when adjusting for the potentially confounding factor of age as covariate.

### Internal reliability

CEBQ subscales demonstrated good to excellent internal consistency (α = 0.82–0.94). MIOH subscales showed acceptable to excellent internal consistency (α = 0.73–0.92) and PSS-4 demonstrated good internal consistency (α = 0.80; Crano & Brewer, 1973; see Supplementary Information for tests).

### Differences in eating behaviours

Significant differences were revealed between groups of children with ASC, ADHD, ASC + ADHD and NT children for food responsiveness, *F*(3,166.914) = 5.938, *p* < .001, η^2^ = .044 (small-medium effect); enjoyment of food, *F*(3,168.057) = 7.602, *p* < .001, η^2^ = .057 (small-medium effect); food fussiness, *F*(3,173.149) = 16.884, *p* < .001, η^2^ = .114 (medium-large effect); and emotional undereating, *F*(3,175.203) = 5.957, *p* < .001, η^2^ = .048 (small-medium effect). The remaining comparisons (emotional overeating, satiety responsiveness and slowness of eating) did not yield significant differences (see Table 3).

**Table 3.** Mean scores, ANOVA and effect size for Children’s Eating Behaviour Questionnaire subscales for children with ASC, ADHD, ASC + ADHD and NT children.

Measures	ASC		ADHD		ASC + ADHD		NT		*F* ^a^ (DoF)	η^2^
	*n*	Mean (*SD*)	*n*	Mean (*SD*)	*n*	Mean (*SD*)	*n*	Mean (*SD*)		
Satiety responsiveness (*N* = 347)	80	2.9 (0.9)	86	3.0 (1.0)	65	2.9 (0.8)	116	2.8 (0.7)	1.225 (3,169.306)	
Slowness in eating (*N* = 345)	80	2.9 (1.0)	86	2.9 (1.3)	64	2.8 (1.2)	115	2.7 (0.8)	1.114 (3,164.809)	
Food fussiness (*N* = 349)	80	3.7 (1.0)	87	3.4 (1.2)	65	3.8 (1.0)	117	2.9 (0.9)	16.884** (3,173.149)	.114
Food responsiveness (*N* = 348)	79	2.7 (1.2)	87	2.9 (1.2)	65	3.0 (1.2)	117	2.4 (0.9)	5.938** (3,166.914)	.044
Enjoyment of food (*N* = 348)	80	3.2 (1.0)	85	3.5 (1.0)	65	3.3 (1.1)	118	3.8 (0.8)	7.602** (3,168.057)	.057
Emotional undereating (*N* = 350)	80	3.3 (0.8)	87	3.3 (0.9)	65	3.4 (0.9)	118	2.9 (0.8)	5.957** (3,175.203)	.048
Emotional overeating (*N* = 345)	79	2.3 (0.9)	85	2.4 (0.9)	65	2.4 (1.0)	116	2.1 (0.8)	2.078 (3,162.962)	

*Note.* N/n varies due to missing data, that is, participants with missing data points in a subscale were excluded from analysis for that subscale. *SD* = standard deviation; η^2^ = eta squared (reported for significant results only); *DoF* *=* degrees of freedom; ASC: children with autism; ADHD: attention deficit hyperactivity disorder.

a Welch’s F (selected due to uneven group sizes and inhomogeneity of variances).

* *p* < .05; **p < .001.

**Figure 1.**
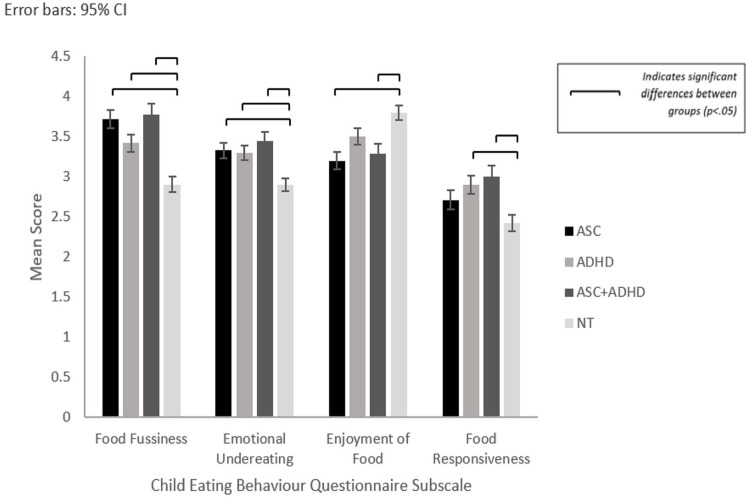
Mean scores for ASC, ADHD, ASC + ADHD and NT groups for Child Eating Behaviour (CEBQ) subscales: Food Fussiness, Emotional Undereating, Enjoyment of Food, and Food Responsiveness with significant differences.

Differences between NDC and NT groups were identified (see Figure 1). No significant differences were found between NDC groups.

### Food fussiness

Significantly higher reported food fussiness was identified for children with ASC (*p* < .001), ADHD (*p* = .004) and ASD + ADHD (*p* < .001) compared to NT children.

### Emotional undereating

All NDC groups reported significantly higher emotional undereating compared to the NT group: ASC (*p* = .012), ADHD (*p* = .036), ASC + ADHD (*p* = .002).

### Enjoyment of food

Significantly lower enjoyment of food was revealed for ASC (*p* < .001) and ASC + ADHD (*p* = .031) compared to the NT group.

### Food responsiveness

Significant differences revealed between ADHD and NT (*p* = .012) and ASC + ADHD and NT (*p* = .003) indicated higher food responsiveness in the ADHD and ASC + ADHD groups compared to NT children.

A significant main effect of group (*p* < .001) remained for all four subscales when accounting for the effects of age and adjusting for multiple comparisons in ANCOVA analysis. Between-group differences identified through exploratory ANOVA Games-Howell post hoc tests were all preserved in ANCOVA Bonferroni-adjusted post hoc tests (see Supplementary Information for results).

### Differences in child mealtime behaviours, family mealtime environments and caregiver stresses and concerns

Significant differences were revealed between groups of families of children with ASC, ADHD, ASC + ADHD and NT children for: problematic child mealtime behaviours, *F*(3,170.615) = 62.348, *p* < .001, η^2^ = .327 (large effect); structure of family meals, *F*(3,168.338) = 20.137, *p* < .001, η^2^ = .137 (medium-large effect); parental concern about child’s diet, *F*(3,166.301) = 14.194, *p* < .001, η^2^ = .100 (medium effect) and spousal stress, *F*(3,147.630) = 27.419, *p* < .001, η^2^ = .203 (large effect). No significant differences were found for the influence of child’s food preferences on what other family members eat. Significant between-group differences were also found relating to caregiver global stress: PSS-4, *F*(3,175.827) = 15.575, *p* < .001, η^2^ = .118 (medium-large effect); and caregiver-reported stress during mealtimes with their child, *F*(3,174.587) = 20.917, *p* < .001, η^2^ = .149 (large effect; see Table 4).

**Table 4.** Group mean scores, standard deviations, ANOVA and effect size for meals in our household subscales, the Perceived Stress Scale-Short Form (PSS-4) and caregiver reported mealtime stress for children with ASC, ADHD, ASC + ADHD and NT children.

Measures	ASC		ADHD		ASC + ADHD		NT		*F* ^a^	η^2^
	*n*	Mean (*SD*)	*n*	Mean (*SD*)	*n*	Mean (*SD*)	*n*	Mean (*SD*)	(DoF)	
MIOH subscale										
Problematic child mealtime behaviours (PMB; *N* = 351)	80	27.6 (10.8)	88	32.7 (12.4)	65	33.6 (11.8)	118	15.9 (9.2)	62.348** (3,170.615)	.327
Influence of child’s food preferences on what other family members eat (IFO; *N* = 287)	65	7.9 (3.5)	70	7.8 (3.2)	54	7.9 (2.9)	98	7.6 (3.1)	0.159 (3,144.884)	
Parental concern about child’s diet (PCD; *N* = 344)	79	19.3 (13.9)	84	17.4 (13.0)	64	19.6 (14.3)	117	9.8 (10.9)	14.194** (3,166.301)	.100
Structure of family meals (SFM; *N* = 349)	79	24.1 (5.1)	88	26.1 (5.9)	64	23.2 (6.1)	118	28.6 (4.5)	20.137** (3,168.338)	.137
Spousal stress (SS; *N* = 313)	73	9.8 (4.3)	76	12.1 (4.5)	54	12.2 (4.6)	110	7.3 (3.7)	27.419** (3,147.630)	.203
PSS-4 (*N* = 349)	80	2.1 (0.6)	88	2.1 (0.7)	65	2.1 (0.8)	116	1.6 (0.7)	15.575** (3,175.827)	.118
Additional items										
Caregiver-reported mealtime stress (*N* = 351)	80	2.2 (0.9)	88	2.5 (1.0)	65	2.5 (1.0)	118	1.6 (0.8)	20.917** (3,174.587)	.149

ASC:; ADHD:; ASCADHD:; NT:; *SD*:; MIOH:; PMB:; IFO:; PCD:; SFM:; SS:; PSS:;

*Note.* N/n varies due to missing data, that is, participant with missing data points in a subscale were excluded from analysis for that subscale. *SD* = standard deviation; η^2^ = eta squared (reported for statistically significant results); *DoF* *=* degrees of freedom; ASC: children with autism; ADHD: attention deficit hyperactivity disorder. *IFO* means calculated for participants who answered all three questions in the scale.

a Welch’s F (selected due to uneven group sizes and inhomogeneity of variances).

** *p* < .001.

**Figure 2.**
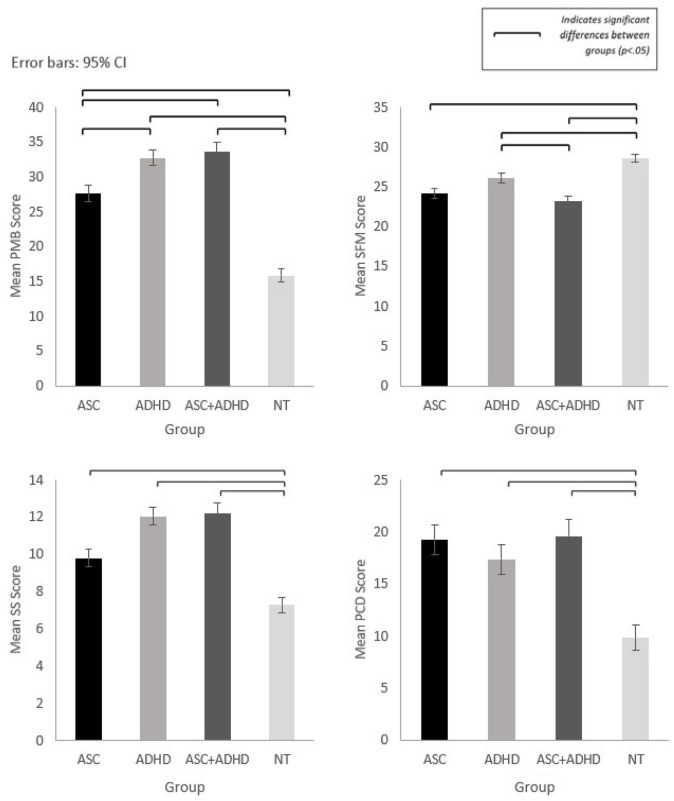
Mean scores for meals in our household subscales with significant differences between groups: Problematic Child Mealtime Behaviours (PMB), Structure of Family Meals (SFM), Parental Concern about Child’s Diet (PCD) and Spousal Stress (SS).

Post hoc analysis revealed differences between NDC and NT groups (see Figure 2).

### Child problematic mealtime behaviour (PMB)

Higher levels of PMB were reported for all NDC groups compared to the NT group (*p* < .001). Differences between NDC groups were also revealed, with ADHD (*p* = .023) and ASC + ADHD (*p* = .011) reporting significantly higher PMB compared to ASC.

### Structure of family mealtimes (SFM)

Significantly lower SFM was revealed for ASC (*p* < .001), ADHD (*p* = .007) and ASC + ADHD (*p* < .001) compared to the NT group, and additionally for ASC + ADHD compared to the ADHD group (*p* = .02).

### Parental concerns about child’s diet (PCD)

Caregivers of NT children reported lower PCD compared to caregivers of children in all NDC groups (*p* < .001).

### Spousal stress related to child’s mealtime behaviour (SS)

Significantly higher spousal stress was reported for all NDC groups compared to the NT group (*p* < .001), and for ASC + ADHD (*p* = .019) and ADHD (*p* = .011) compared to ASC.

### Caregiver stress

Caregivers in all NDC groups reported feeling stressed by their child’s mealtime behaviour significantly more often than parents of NT children (*p* < .001).

### Perceived Stress Scale (PSS-4)

Global stress levels were significantly higher for all NDC groups compared to the NT group (*p* < .001).

A significant main effect of group (*p* < .001) was retained for all measures after adjusting for the effect of age and multiple comparisons through ANCOVA analysis. All between-group differences were preserved in ANCOVA Bonferroni-adjusted post hoc tests (see Supplementary Information for results).

### Summary of eating and mealtime behavioural profile by group

*All NDC groups* reported significant differences compared to families of NT children relating to less structured family meals and higher food fussiness, emotional undereating, problematic mealtime behaviour, parental concern about child’s diet, caregiver global stress, spousal stress and caregiver-reported stress during mealtimes with their child.

*ADHD group* additionally reported significantly higher food responsiveness compared to NT children and significantly higher problematic mealtime behaviour and spousal stress compared to the ASC group.

*ASC group* reported significantly lower enjoyment of food compared to the NT group.

*ASC + ADHD group* reported significantly lower enjoyment of food and higher food responsiveness compared to NT children. Significantly higher problematic mealtime behaviour and spousal stress were reported compared to the ASC group, with less-structured family meals compared to the ADHD group.

## Discussion

This study provides the first comprehensive profile of eating and mealtime characteristics of children aged 3–15 years with ASC, ADHD and ASC + ADHD, compared to NT children. The novel findings reveal that children with these NDC and their families face some shared and some unique differences at mealtimes compared to NT families, and to each other. This study indicates an additive profile of more extreme eating and mealtime behaviours in children with ASC + ADHD compared to those with ASC, ADHD and NT children.

### Eating behaviour

Higher food selectivity (referred to as food fussiness, FF) and emotional undereating (EUE) were reported for children with NDC compared to NT children. This corroborates previous findings of elevated levels of FF in autistic and ADHD compared to NT populations, extending this to a broader age range and to children with ASC + ADHD (Dovey et al., 2019; Smith et al., 2020).

Building on prior research reporting elevated EUE in clinical autistic and non-clinical ADHD samples (Harris et al., 2022; Tong et al., 2017; Wallace et al., 2021), this study suggests EUE may also be prevalent in children with ADHD and ASC + ADHD. Emotional eating is linked to emotional regulation differences in both ASC and ADHD, although is not included in the *DSM-5* diagnostic criteria (Cibralic et al., 2019; el Archi et al., 2020). Emotional regulation differences affect parental feeding and coping strategies (Craig et al., 2020; Harris et al., 2020; Steinsbekk et al., 2018), while emotional eating correlates with less cohesive family functioning (Moen et al., 2016). Considered together, these findings suggest a gene-environment interaction, highlighting the need for a deeper understanding of emotional eating and the broader familial context for NDC families.

Significantly lower enjoyment of food (EF) was reported for autistic children and those with ASC + ADHD, while significantly higher food responsiveness (FR) was reported for children with ADHD and ASC + ADHD compared to NT children. FR and EF scales assess general appetite and desire to eat, with high scores indicating ‘food approach’ and low scores indicating ‘food avoidant’ behaviours (Carnell & Wardle, 2007). These results support research suggesting an association between autistic features and food avoidance behaviours (Nadeau et al., 2022) and higher levels of food approach behaviours in children with ADHD (Leventakou et al., 2022).

Importantly, this study advances understanding of children with ADHD and dual ASC + ADHD diagnoses, for whom food approach *and* food avoidance were reported, with more elevated FF, EUE, FR and EF behaviours compared to the NT group. These findings may indicate dysregulated eating associated with the ASC + ADHD phenotype, characterised by increased food responsiveness to a restricted range of lower quality foods (Harris et al., 2022). Indeed, the undetected co-occurrence of ADHD and ASC could provide an explanation for Nadeau and colleagues’ (2022) findings of the ‘selective overeating’ phenotype, combining food approach and avoidance, reported in autistic children. ADHD traits may also contribute to increased appetite and responsivity to food cues (Harris et al., 2022). The seemingly contradictory profiles of food approach and food avoidance in ADHD and ASC + ADHD may be explained by ‘impulsivity’ and ‘inattention’ traits (Harris et al., 2020), suggesting a cumulative effect and supporting the theory of additivity (Hollingdale et al., 2020).

In contrast to some studies (Fuemmeler et al., 2020; Gorgu, 2020; Öz & Bayhan, 2021), this research did not find differences between NDC and NT groups for satiety responsiveness (SR), slowness in eating (SE) or emotional overeating (EOE). Inconsistent EOE findings may result from scales not discriminating between positive and negative EOE (Manchón et al., 2021). While Harris and colleagues (2022) found SE and SR were associated with autistic traits and EOE with ADHD traits, they did not compare clinical groups and subclinical traits may not accurately represent children with clinical diagnoses.

### Mealtime environment

Differences in mealtime experiences were identified for all NDC groups compared to the NT group. Significantly higher child mealtime behaviours perceived to be ‘problematic’ (PMB), dietary concerns (PCD), global caregiver stress, mealtime-related stress, spousal stress (SS) and less structured mealtime environments (SFM) were reported for all NDC groups compared to NT children. Increased PMB has been associated with food selectivity in children with NDC and may correlate with heightened anxiety and avoidance of non-preferred foods (Curtin et al., 2015; Farrow & Coulthard, 2012). The elevated levels of food selectivity in all NDC groups may contribute to the increased behavioural differences identified in this study.

Using MIOH to assess mealtime behaviours in children with ADHD and ASC + ADHD, this study found significantly higher levels of perceived PMB in these groups compared to those with an ASC only diagnosis, offering novel insights building on limited prior research to suggest that mealtime experiences for families of children with ADHD and ASC + ADHD may be more challenging and pervasive than previously understood (Ciesielski et al., 2020; Moen et al., 2016). Exploring the association between these behaviours and specific features of ADHD including hyperactivity, impulsivity and emotional regulation differences may be beneficial (Young et al., 2020).

In contrast to studies focusing solely on child eating behaviours (Kaisari et al., 2017; Ledford et al., 2018; Marí-Bauset et al., 2014), this research explored the impact of eating and mealtime behaviours on family systems. Families of children with NDC reported greater divergence from conventional models of mealtimes and higher levels of dietary concern than families of NT children. In addition, greater levels of caregiver and spousal mealtime stress were reported in all NDC groups. This corroborates prior research comparing the stress experienced at mealtimes for caregivers of autistic and NT children (Crowe et al., 2016). Although currently underrepresented in the empirical literature, caregiver and spousal reported stress were significantly higher in the ADHD and ASC + ADHD groups compared to the ASC group, highlighting the potentially unaddressed needs of these populations. Differences were not found between NDC and NT groups in terms of influence of child’s food preferences on what other family members eat (IFO) in contrast to Curtin et al. (2015) but supporting and extending findings from the initial MIOH validation study comparing autistic children to NT children aged 3–11 to a wider age range (Anderson et al., 2012).

Heritability of ASC and ADHD emphasises the potential presence of neurodivergent traits in multiple family members (Chen et al., 2017; Miller et al., 2019), highlighting the need to consider the broader familial context and the role of other family members when examining eating and mealtime behaviours (Curtiss & Ebata, 2019; Moen et al., 2016). While previous research suggests that shared family meals and the quality of mealtime structure may act as protective factors for children’s physical and mental health, the mechanisms for these effects remain unclear (Snuggs & Harvey, 2023). Family connectedness might serve as an explanation (Hammons & Fiese, 2011), with a cohesive family environment positively associated with child development (Mendes-Sousa et al., 2023). Our findings indicate that caregivers of children, particularly those in the ASC + ADHD group, may experience elevated levels of stress, which in turn may affect child development and internalising and externalising behaviours (Jones et al., 2021). Therefore, it becomes crucial to explore whether shared mealtimes contribute to increased stress or conflict, especially if enforced conventions such as table manners and sharing the same foods may have varying effects on different families. In addition, future research should investigate whether parents do indeed perceive these mealtime behaviours as ‘problematic’ as this perception could affect health and wellbeing outcomes.

Collectively, these findings highlight that while there are some similarities with families of NT children, eating and mealtime experiences in families of children with NDC are demonstrably different. They suggest that eating difficulties may persist beyond what may be regarded as the typical developmental trajectory in children with NDC (Taylor & Emmett, 2019). More elevated differences in terms of perceived ‘problematic’ child mealtime behaviours and caregiver stress associated with families of children with ADHD and ASC + ADHD are clinically noteworthy and intervention and support may be particularly helpful for these families (Young et al., 2020). Currently, the most commonly adopted mealtime support interventions have focussed on increasing the food volume intake, diet quality and repertoire of autistic children (Ledford et al., 2018; Marshall et al., 2014; Sarcia, 2020). Behavioural and sensory-based interventions, food chaining and parent-training programmes developed to specifically target food selectivity in autistic children have limited proven effectiveness (Breda et al., 2024), while mealtime support for children with ADHD is scarce (Ciesielski et al., 2020; Moen et al., 2016; Thorsteinsdottir et al., 2021). Our findings suggest the need for a more nuanced approach to interventions that goes beyond food intake, instead taking into consideration the broader familial environment, including parental stress and child mealtime behaviours, and the interaction of reported eating differences for children with NDC (Gent et al., 2024). To address these complexities, a transdiagnostic, holistic and needs-led approach to professional mealtime support for families of children with NDC is recommended.

### Future research

This study illustrates the importance of recognising eating and mealtime behaviours within the broader context of complex bio-psychosocial factors and the need to further understand their interplay (Moal et al., 2021; Neumark-Sztainer et al., 2013). Future research employing longitudinal designs with multiple, repeated measures should explore how these behaviours and environments evolve over the developmental trajectory for families of children with NDC (Harris et al., 2020). The study also calls for efforts to develop a shared understanding of mealtimes, creating measures capturing both universal and neurodivergent familial experiences in co-creation with NDC families, incorporating a strengths-based approach (Gent et al., 2024).

### Limitations

Several limitations are acknowledged. Despite efforts to recruit from underrepresented groups, a high percentage of respondents were female and white. While mothers of children with NDC may often adopt primary caregiver responsibilities associated with mealtimes and feeding challenges (McAuliffe et al., 2019), there is growing recognition of the need to incorporate the perspectives of fathers, siblings and the child to better understand their contribution to the family feeding environment (Harcourt & Einarsdottir, 2011; Harris et al., 2020; Nilsson et al., 2015) as well as to elevate neurodivergent voices (Bottema-Beutel et al., 2021). Lack of ethnic diversity in the sample may affect generalisability of study findings and suggests that more effective strategies are needed to engage these groups in future neurodevelopmental research (Ratto et al., 2017).

Presentation and phenotypic behaviour may differ by gender in ASC and ADHD, and limited research suggests that this may also apply to some eating behaviours, although heterogeneous findings have been reported to date (Kaisari et al., 2017; van’t Hof et al., 2020; Wallace et al., 2021). The male gender skew of the child sample is a limitation common to neurodevelopmental research, and studies to date have predominantly looked at male samples (> 80%; Baraskewich et al., 2021). Exploring age and gender differences would provide a more comprehensive understanding of mealtime experiences in families of children with NDC. Attempts to recruit more balanced numbers of males and females should be prioritised (Slobodin & Davidovitch, 2019).

This study is based on caregiver report and no direct observations were conducted due to the nature and breadth of the topics researched. Online administration facilitated recruitment of a large sample, providing confidence in the generalisability and ecological validity of the findings (Cascio et al., 2021; Cohen, 1992). While the study aimed to investigate the differences and difficulties in eating and mealtime experiences, it acknowledges the importance of future research in exploring positive dimensions and enablers that contribute to mealtime enjoyment and familial wellbeing.

## Conclusion

This is the first study to examine child eating and mealtime behaviour and family mealtime experiences across under-researched NDC populations compared to NT children and their families. Use of participatory Think Aloud methods, recruitment of a large sample across a wide age range and a comprehensive set of measures with excellent internal consistency in ASC + ADHD and ADHD populations, contribute to a holistic representation of family mealtime characteristics in neurodivergent households. The findings from this study highlight the distinct eating and mealtime experiences in NDC families. They emphasise the importance of adopting a bio-psychosocial approach centred on the whole family to further understand the impact and causal factors of these behaviours and experiences, and a transdiagnostic approach to mealtime support, with strategies tailored to meet the needs of distinct NDC profiles.

## Supplemental Material

sj-docx-1-aut-10.1177_13623613241277605 – Supplemental material for Comparing eating and mealtime experiences in families of children with autism, attention deficit hyperactivity disorder and dual diagnosis Supplemental material, sj-docx-1-aut-10.1177_13623613241277605 for Comparing eating and mealtime experiences in families of children with autism, attention deficit hyperactivity disorder and dual diagnosis by Zoe Matthews, Donna Pigden-Bennett, Teresa Tavassoli and Sarah Snuggs in Autism

sj-docx-2-aut-10.1177_13623613241277605 – Supplemental material for Comparing eating and mealtime experiences in families of children with autism, attention deficit hyperactivity disorder and dual diagnosis Supplemental material, sj-docx-2-aut-10.1177_13623613241277605 for Comparing eating and mealtime experiences in families of children with autism, attention deficit hyperactivity disorder and dual diagnosis by Zoe Matthews, Donna Pigden-Bennett, Teresa Tavassoli and Sarah Snuggs in Autism

sj-docx-3-aut-10.1177_13623613241277605 – Supplemental material for Comparing eating and mealtime experiences in families of children with autism, attention deficit hyperactivity disorder and dual diagnosis Supplemental material, sj-docx-3-aut-10.1177_13623613241277605 for Comparing eating and mealtime experiences in families of children with autism, attention deficit hyperactivity disorder and dual diagnosis by Zoe Matthews, Donna Pigden-Bennett, Teresa Tavassoli and Sarah Snuggs in Autism

sj-docx-4-aut-10.1177_13623613241277605 – Supplemental material for Comparing eating and mealtime experiences in families of children with autism, attention deficit hyperactivity disorder and dual diagnosis Supplemental material, sj-docx-4-aut-10.1177_13623613241277605 for Comparing eating and mealtime experiences in families of children with autism, attention deficit hyperactivity disorder and dual diagnosis by Zoe Matthews, Donna Pigden-Bennett, Teresa Tavassoli and Sarah Snuggs in Autism

sj-docx-5-aut-10.1177_13623613241277605 – Supplemental material for Comparing eating and mealtime experiences in families of children with autism, attention deficit hyperactivity disorder and dual diagnosis Supplemental material, sj-docx-5-aut-10.1177_13623613241277605 for Comparing eating and mealtime experiences in families of children with autism, attention deficit hyperactivity disorder and dual diagnosis by Zoe Matthews, Donna Pigden-Bennett, Teresa Tavassoli and Sarah Snuggs in Autism

## Notes

1. Nomenclature to best reflect the preferences of the autistic community has been adopted where possible (Kenny et al., 2016).
