# Supplementary material for: Comparing eating and mealtime experiences in families of children with autism, attention deficit hyperactivity disorder and dual diagnosis
Source: Autism. 2024 Sep 12;29(2):518–35. doi: 10.1177/13623613241277605 (PMC11816458; doi:10.1177/13623613241277605)
Supplement: sj-docx-1-aut-10.1177_13623613241277605 – Supplemental material for Comparing eating and mealtime experiences in families of children with autism, attention deficit hyperactivity disorder and dual diagnosis [file sj-docx-1-aut-10.1177_13623613241277605.docx]

**Supplementary Information**

*Think-Aloud Participant Demographics*

| Participant Number | Age  Group | Gender | Caregiver status | Highest  Educational level | Child  diagnostic status | Child  age | Child  sex |
| --- | --- | --- | --- | --- | --- | --- | --- |
| 1 | 30-39 | F | Single parent | Higher education | ASD | 10 | M |
| 2 | 50-59 | M | Dual parent | Further education | ADHD | 14 | M |
| 3 | 40-49 | F | Dual parent | Further education | ADHD | 13 | M |
| 4 | 40-49 | F | Dual parent | Further education | ADHD | 9 | F |
| 5 | 50-59 | F | Single parent | Higher education | ASD+ADHD | 15 | F |
| 6 | 40-49 | F | Dual parent | Postgraduate degree | ASD+ADHD | 12 | F |
| 7 | 40-49 | F | Dual parent | Further education | TD | 9 | F |
| 8 | 50-59 | M | Dual parent | Higher education | TD | 7 | M |
| 9 | 40-49 | M | Dual parent | Postgraduate degree | TD | 6 | F |

*Note.* F= female; M = male; Highest education level achieved was taken as a proxy for socio-economic status (Baker, 2014).
