# Supplementary material for: Comparing eating and mealtime experiences in families of children with autism, attention deficit hyperactivity disorder and dual diagnosis
Source: Autism. 2024 Sep 12;29(2):518–35. doi: 10.1177/13623613241277605 (PMC11816458; doi:10.1177/13623613241277605)
Supplement: sj-docx-2-aut-10.1177_13623613241277605 – Supplemental material for Comparing eating and mealtime experiences in families of children with autism, attention deficit hyperactivity disorder and dual diagnosis [file sj-docx-2-aut-10.1177_13623613241277605.docx]

## Supplementary Information

*Defined Problems, Implemented Solutions from Think-Aloud Study and Intended Outcomes for Study*

| Questionnaire subscale(s) / section(s) | Item | Issues | Participant group affected | Changes made to survey | Intended outcomes for study |
| --- | --- | --- | --- | --- | --- |
| Demographics  MIOH all subscales  CEBQ all subscales | Medication | Participants gave either polarised responses or middle ‘on the fence’ responses depending on whether they answered questions assuming child is medicated or not medicated. | ADHD / ADHD+ASC | Included additional information at the point of asking participants about whether or not their child took medication relating to their diagnosis to answer on the basis that their child was taking medication. | Improve understanding and clarity of participant response required |
| Demographics | Clinical diagnosis | Participants with suspected but unconfirmed diagnosis (e.g., on the diagnostic pathway) were unclear which option to select in answer to the question: “Does your child have a clinical diagnosis for ADHD / ASC / combined ADHD and ASC?” | ASC / ADHD / ADHD+ASC | Amended wording of question to “**confirmed** clinical diagnosis” | Increased confidence that participants will self-select the correct diagnostic group, which will in turn give more confidence to the results. |
| MIOH Structure of Family Meals | The television is on in the same room when my child is eating meals. | Participants flagged the problem that use of screens other than TVs (e.g., handheld electronic devices) was not covered – leading to confusion over how this should be answered. | ASC, ADHD, ASC+ADHD, NT | Changed statement to: “Screen (e.g. iPad) or television is on when my child is eating meals.’” | Bringing the survey up to date and relevant for a modern UK audience. |
| MIOH Structure of Family Meals | We say grace or have a ritual at the start of meals. | Participants questioned content | ADHD, NT | Amended wording of item to: “We have a ritual (e.g., saying grace) at the start of meals.” | Aiming to improve relevance for a modern UK audience |
| MIOH Problematic Child Mealtime Behaviour | Question rubric / Parental Perception of Problem questions | Long and unclear question rubric leads to inconsistent answers from participants to the question of “for each statement, please choose how much of a problem that aspect of your child’s behaviour is for you.” | ASC, ADHD, NT | Shortening and rephrasing of question rubrics to ensure consistency and avoid confusion throughout the survey. | Improving accuracy, quality and relevance of participant responses |
| MIOH Problematic Child Mealtime Behaviour | My child refuses to come when it is time to eat | Item does not capture nuance of the issue experienced by parents in these populations – more common problem that children do not come willingly when it is time to eat. | ASC, ADHD, ASC+ADHD | Updated the statement to: “My child refuses to come or does not come willingly when it is time to eat.” | Capturing nuance and making this relevant for problems experienced by neurodiverse populations. |
| MIOH Problematic Child Mealtime Behaviour | My child complains about what is served. | Item does not capture nuance of the issue experienced by parents in these populations – parents often adapt to the child and tend only to serve what they know they will eat. | ASC, ADHD, ASC+ADHD, NT | Introduction of new item:  “My child is only served food that l know they will eat.” With 5-point Likert scale rating 0=never, 4=always. | Improving relevance/suitability for current UK and clinical audience |
| MIOH Problematic Child Mealtime Behaviour | My child has tantrums or acts out during meals. | Item is appropriate for a younger audience but ‘tantrums’ and ‘acts out’ questioned by parents of older children, who struggled to respond to this item. | ADHD, ASC+ADHD  >12-year-olds | Switched order of items in statement and rephrased to: “’My child plays up or has tantrums during meals.’ | Making sure scale is using vocabulary appropriate for a UK audience and for older children. |
| MIOH Problematic Child Mealtime Behaviour | My child has poor table manners. | Several parents struggled to respond and questioned what good table manners looks for younger children. | ADHD, NT | Changed item to: “My child has poor table manners **for their age**.” To give a relative comparator, so responses reflect child behaviour in line with parent expectation. | Aiming to improve accuracy, quality and relevance of participant responses |
| Usability | Question rubrics | Wordy and inconsistent phrasing of rubrics across questionnaires, including different subscales refer to different time periods | ADHD, NT | Made all time periods consistently one month – longer recall periods are more likely to produce less accurate reported estimates (Stull et al., 2009, Bhandari and Wagner, 2006) and wording made consistent across rubrics without changing meanings. | Improving accuracy, quality and relevance of participant responses, user experience/usability |
| General | Question rubrics | Participants answering about breakfast and lunchtimes– i.e., not clear enough that the focus of the overall survey is on evening mealtimes. | ASC, ADHD, ASC+ADHD, NT | Updated the simplified question rubric to specify ‘evening mealtimes in your household’ to provide more clearer context for participants. | Improving accuracy, quality and relevance of participant responses |
| Usability | Order of survey | Some confusion about whether answering about child or themselves (parent) when moving between demographics and subscales. | ASC, ADHD, ASC+ADHD, NT | Changed order of survey. | Improving user experience and usability |
| Usability | MIOH IFO and SS subscale response options | Instruction to skip question if circumstances didn’t apply to participant (e.g., no partner/spouse, no other children in household) caused confusion over response/was missed and middle option of Likert scale selected. | ADHD, NT | Added in ‘n/a’ answer option and updated question rubric for both subscales. | Improving quality of participant responses and improving user experience |
| Content | New questions added (quantitative) | Parents report on spousal stress in MIOH SS subscale and global stress in PSS-4 items, but no quantitative measure to capture their own mealtime experiences with their child. | ASC+ADHD, NT | Added: “You have felt enjoyment when eating with your child.” And “You have felt stressed by your child’s mealtime behaviour.” Alongside PSS-4 items. | Improving nuance and adding depth to data collection regarding parental mealtime experiences |
| SWAN | Ignores extraneous stimuli | Participants paused over responses/ repeated multiple times/ questioned content and didn’t understand intended meaning. | ASC, ADHD, ASC+ADHD, NT | Changed to: “Ignores distractions (extraneous stimuli, e.g. outside noises or talking).” | Improving accessibility and quality of participant responses whilst maintaining intended meaning |
| SWAN | Modulates motor activity (inhibits inappropriate running or climbing) | Participants paused over responses/ repeated multiple times/ questioned content and didn’t understand intended meaning. | ADHD, ASC+ADHD, NT | Changed phrasing and switched word order to: “Holds back from inappropriate running or climbing (modulates motor activity).” | Improving accessibility and quality of participant responses whilst maintaining intended meaning |
| SWAN | Settles down and rests (controls constant activity) | Wording flagged as less appropriate for older children. | ASC+ADHD, ADHD | Switched word order to: “Controls constant activity (settles down and rests). | Improving accessibility and quality of participant responses whilst maintaining intended meaning |
| SWAN | Modulates verbal activity (controls excessive talking) | Participants paused over responses/ repeated multiple times/ questioned content and didn’t understand intended meaning. | ADHD, ASC+ADHD, NT | Switched word order to: “Controls excessive talking (modulates verbal activity).” | Improving accessibility and quality of participant responses whilst maintaining intended meaning |
| SWAN | Reflects on questions (controls blurting out answers). | Participants paused over responses/ repeated multiple times. | ASC+ADHD, NT | Switched word order to: Controls blurting out answers (reflects on questions). | Improving accessibility and quality of participant responses whilst maintaining intended meaning |
| Usability | AQ-10 Child and Adolescent question rubric | Participants did not read instructions in question rubric and answered the inappropriate AQ-10 (Child or Adolescent versions) for their child’s age. | ADHD, NT | Added new question asking parent to confirm if child under 12, automatically directs them to appropriate AQ-10 scale for their child’s age. | Improving usability and user experience |
| AQ10 | AQ-10 Child  AQ-10 Adol | Researchers identified statements contained pronouns presented in binary he/she terms. | ASC, ADHD, ASC+ADHD, NT | Pronouns changed from he/she to they/them. | Improve inclusivity |
