# Supplementary material for: Comparing eating and mealtime experiences in families of children with autism, attention deficit hyperactivity disorder and dual diagnosis
Source: Autism. 2024 Sep 12;29(2):518–35. doi: 10.1177/13623613241277605 (PMC11816458; doi:10.1177/13623613241277605)
Supplement: sj-docx-3-aut-10.1177_13623613241277605 – Supplemental material for Comparing eating and mealtime experiences in families of children with autism, attention deficit hyperactivity disorder and dual diagnosis [file sj-docx-3-aut-10.1177_13623613241277605.docx]

**Supplementary Information**

*Caregiver Demographics*

| Demographics | ASC (*n*=80) | ADHD (*n*=88) | ASC+ADHD (*n*=65) | NT (*n*=118) |
| --- | --- | --- | --- | --- |
| **Age range in years (%)** |  |  |  |  |
| 16-20 | 1 (1.3) | 0 | 0 | 0 |
| 20-29 | 4 (5.0) | 6 (6.8) | 4 (6.2) | 4 (3.4) |
| 30-39 | 28 (35.0) | 41 (46.6) | 26 (40.0) | 41 (34.7) |
| 40-49 | 37 (46.3) | 33 (37.5) | 24 (36.9) | 59 (50.0) |
| 50-59 | 10 (12.5) | 7 (8.0) | 10 (15.4) | 14 (11.9) |
| 60-69 | 0 | 1 (1.1) | 1 (1.5) | 0 |
| **Mean Age (*SD*)*** | 40.9 (8.0) | 39.5 (7.9) | 41.1 (8.7) | 41.5 (7.2) |
| **Relationship to child (%)** |  |  |  |  |
| Mother | 73 (91.3) | 85 (96.6) | 61 (93.8) | 109 (93.2) |
| Father | 6 (7.5) | 2 (2.3) | 2 (3.1) | 8 (6.8) |
| Carer | 1 (1.3) | 0 | 0 | 0 |
| Stepmother | 0 | 1 (1.1) | 1 (1.5) | 0 |
| Grandmother | 0 | 0 | 1 (1.5) | 0 |
| **Gender (%)** |  |  |  |  |
| Male | 6 (7.5) | 2 (2.3) | 2 (3.1) | 9 (7.6) |
| Female | 72 (91.3) | 86 (97.7) | 61 (96.9) | 109 (92.4) |
| Other | 1 (1.3) | 0 | 0 | 0 |
| **Ethnicity (%)** |  |  |  |  |
| White | 73 (91.3) | 83 (94.3) | 65 (100) | 110 (93.2) |
| Asian / Asian British | 0 | 3 (3.4) | 0 |  |
| Black / Black British | 0 | 1 (1.1) | 0 | 1 (0.8) |
| Mixed / multiple ethnic groups | 5 (6.3) | 0 | 0 | 3 (2.5) |
| Other | 2 (2.5) | 0 | 0 | 1 (0.8) |
| Prefer not to say | 0 | 1 (1.1) | 0 | 2 (1.7) |
| **Household structure (%)** |  |  |  |  |
| Dual parent | 66 (83.5) | 75 (85.2) | 45 (71.4) | 105 (89.0) |
| Single parent | 10 (12.7) | 13 (14.8) | 16 (25.4) | 10 (8.5) |
| Carer | 1 (1.3) | 0 | 1 (1.6) | 0 |
| Other | 1 (1.3) | 0 | 1 (1.6) | 3 (2.5) |
| **Highest level of education completed (%)** |  |  |  |  |
| Secondary education | 5 (6.3) | 4 (4.5) | 5 (7.7) | 7 (5.9) |
| Further education | 32 (40.0) | 26 (29.5) | 18 (27.7) | 23 (19.5) |
| Higher education | 20 (25.0) | 31 (35.2) | 31 (47.7) | 47 (39.8) |
| Postgraduate education | 22 (27.5) | 24 (27.3) | 10 (15.4) | 40 (33.9) |
| Prefer not to say | 1 (1.3) | 0 | 1 (1.5) | 1 (0.8) |

*Calculated using median scores of participants’ age ranges.

*Child Demographics*

| Demographic | ASC (*n*=80) | ADHD (*n*=88) | ASC+ADHD (*n*=65) | NT (*n*=118) |
| --- | --- | --- | --- | --- |
| **Mean age (*SD*)** | 9.6 (3.2) | 9.3 (3.1) | 9.9 (2.8) | 8.6 (3.9) |
| **Gender (%)** |  |  |  |  |
| Male | 56 (70.0) | 72 (81.8) | 56 (86.2) | 66 (55.9) |
| Female | 23 (28.7) | 16 (18.2) | 7 (10.8) | 52 (44.1) |
| Non-binary | 1 (1.3) | 0 | 0 | 0 |
| Prefer not to say | 0 | 0 | 1 (1.5) | 0 |
| Other | 0 | 0 | 1 (1.5) | 0 |
| **Ethnicity (%)** |  |  |  |  |
| White | 74 (92.5) | 78 (88.6) | 63 (96.9) | 106 (89.9) |
| Asian / Asian British | 0 | 3 (3.4) | 0 | 1 (0.8) |
| Black / Black British | 1 (1.3) | 1 (1.1) | 0 | 0 |
| Mixed / Multiple ethnic groups | 3 (3.8) | 4 (4.5) | 1 (1.5) | 9 (7.6) |
| Other | 1 (1.3) | 0 | 1 (1.5) | 1 (0.8) |
| Prefer not to say | 0 | 1 (1.1) | 0 | 1 (0.8) |
| **Medication status ^a^ (%)** |  |  |  |  |
| Taking medication relating to their primary clinical diagnosis | 12 (15.2) | 37 (42.0) | 35 (55.6) |  |
| Not taking medication relating to their primary clinical diagnosis | 60 (75.9) | 30 (34.1) | 21 (33.3) |  |
| Not known | 7 (8.9) | 21 (23.9) | 7 (11.1) |  |
| **Type of medication^a^ (%)** |  |  |  |  |
| Stimulant medication | 0 | 33 (37.5) | 27 (42.9) |  |
| Non-stimulant / other medication | 12 (15.2) | 4 (4.6) | 7 (11.1) |  |
| **Co-occurring/secondary diagnoses (%)** |  |  |  |  |
| One or more other diagnoses | 33 (41.8) | 27 (30.7) | 38 (60.3) | 12 (10.2) |
| No other diagnoses | 37 (46.8) | 49 (55.7) | 20 (31.7) | 95 (80.5) |
| Anxiety Disorder (AD) | 12 (15.2) | 8 (9.1) | 14 (22.2) | 3 (2.5) |
| Conduct Disorder (CD) | 0 | 2 (2.3) | 1 (1.6) | 0 |
| Depression | 1 (1.3) | 1 (1.3) | 1 (1.6) | 1 (0.8) |
| Dyslexia | 5 (6.3) | 6 (6.8) | 7 (11.1) | 9 (7.6) |
| Dyspraxia | 3 (3.8) | 5 (5.7) | 6 (9.5) | 1 (0.8) |
| Eating Disorder (ED) | 1 (1.3) | 1 (1.1) | 1 (1.6) | 0 |
| Epilepsy | 1 (1.3) | 0 | 2 (3.2) | 0 |
| Obsessive Compulsive Disorder (OCD) | 2 (2.5) | 1 (1.1) | 3 (4.8) | 0 |
| Oppositional Defiant Disorder (ODD) | 0 | 5 (5.7) | 3 (4.8) | 0 |
| Pathological Demand Disorder (PDD) | 3 (3.8) | 2 (2.3) | 9 (14.3) | 0 |
| Sensory Processing Disorder | 13 (16.5) | 5 (5.7) | 16 (25.4) | 1 (0.8) |
| Tourette’s Syndrome (TS) | 1 (1.3) | 2 (2.3) | 2 (3.2) | 0 |
| Other | 7 (8.9) | 7 (8.0) | 3 (4.8) | 1 (0.8) |
| **Dietary restrictions (%)** | 14 (17.7) | 13 (14.8) | 13 (20.6) | 9 (7.6) |
| **Mean number of siblings living at home with child (*SD*)** | 2.0 (0.8) | 2.1 (0.8) | 2.1 (0.7) | 2.0 (0.7) |

**^a^** Calculated for ASC, ADHD and ASC+ADHD groups only.
