# Supplementary material for: Comparing eating and mealtime experiences in families of children with autism, attention deficit hyperactivity disorder and dual diagnosis
Source: Autism. 2024 Sep 12;29(2):518–35. doi: 10.1177/13623613241277605 (PMC11816458; doi:10.1177/13623613241277605)
Supplement: sj-docx-4-aut-10.1177_13623613241277605 – Supplemental material for Comparing eating and mealtime experiences in families of children with autism, attention deficit hyperactivity disorder and dual diagnosis [file sj-docx-4-aut-10.1177_13623613241277605.docx]

**Supplementary Information**

*Reliability Analysis of Final Questionnaire Measures with Reported Cronbach’s Alpha (α) and Threshold Level.*

| Measure | Cronbach’s Alpha | Level * |
| --- | --- | --- |
| Children’s Eating Behaviour Questionnaire |  |  |
| Food Fussiness | .94 | Excellent |
| Enjoyment of Food | .94 | Excellent |
| Food Responsiveness | .91 | Excellent |
| Slowness in Eating | .88 | Good |
| Satiety Responsiveness | .84 | Good |
| Emotional Undereating | .82 | Good |
| Emotional Overeating | .82 | Good |
| Meals In Our Household |  |  |
| Problematic Child Mealtime Behaviour | .92 | Excellent |
| Parental Concern about Child’s Diet | .91 | Excellent |
| Spousal Stress Related to Child’s Mealtime Behaviours | .9 | Excellent |
| Influence of Child’s Preferences on What Others Eat | .81 | Good |
| Structure of Family Meals | .73 | Acceptable |
| Perceived Stress Scale – Short Form PSS-4 | .8 | Good |

* (Field, 2020)
