# Supplementary material for: Comparing eating and mealtime experiences in families of children with autism, attention deficit hyperactivity disorder and dual diagnosis
Source: Autism. 2024 Sep 12;29(2):518–35. doi: 10.1177/13623613241277605 (PMC11816458; doi:10.1177/13623613241277605)
Supplement: sj-docx-5-aut-10.1177_13623613241277605 – Supplemental material for Comparing eating and mealtime experiences in families of children with autism, attention deficit hyperactivity disorder and dual diagnosis [file sj-docx-5-aut-10.1177_13623613241277605.docx]

**Supplementary Information**

*Estimated Marginal Means, Standard Error, ANCOVA with Age as Covariate and Effect Sizes for CEBQ Subscales for ASC, ADHD, ASC+ADHD and NT children.*

| Measures | ASC | |  | ADHD | |  | ASC+ ADHD | |  | NT | |  | Group | |  | Age | |
| --- | --- | --- | --- | --- | --- | --- | --- | --- | --- | --- | --- | --- | --- | --- | --- | --- | --- |
|  | *n* | _Adj_*M*  (*SE*) |  | *n* | _Adj_*M*  (*SE*) |  | *n* | _Adj_*M*  (*SE*) |  | *n* | _Adj_*M*  (*SE*) |  | *F* (DoF) | *ηp²* |  | *F* (DoF) | *ηp²* |
| Food fussiness (*N*=349) | 80 | 3.7 (0.11) |  | 87 | 3.4 (0.11) |  | 65 | 3.8 (0.13) |  | 117 | 2.9 (0.09) |  | 16.560** (3,344) | .126 |  | 7.620* (1,344) | .022 |
| Food responsiveness (*N*=348) | 79 | 2.7 (0.12) |  | 87 | 2.9 (0.12) |  | 65 | 3.1 (0.13) |  | 117 | 2.4 (0.10) |  | 6.572** (3,343) | .054 |  | 10.847* (1,343) | .031 |
| Enjoyment of food (*N*=348) | 80 | 3.2 (0.11) |  | 85 | 3.5 (0.11) |  | 65 | 3.4 (0.12) |  | 118 | 3.8 (0.09) |  | 6.622** (3,343) | .055 |  | 0.208 (1,343) |  |
| Emotional undereating (*N*=350) | 80 | 3.3 (0.09) |  | 87 | 3.5 (0.11) |  | 65 | 2.9 (0.08) |  | 118 | 2.9 (0.8) |  | 7.214** (3,345) | .059 |  | 9.792* (1,345) | .028 |

Notes

*N/n* varies due to missing data. i.e., participants with missing data points in a subscale were excluded from analysis for that subscale.

*significant at *p*<.05; **significant at *p*<.001.

ANCOVA calculated for significant ANOVA subscales only.

*M* = mean; _Adj_*M* = estimated marginal mean, *SE* = standard error; *ηp²* = partial eta squared (reported for significant results only); *DoF =* degrees of freedom.

*Estimated Marginal Means, Standard Error, ANCOVA with Age as Covariate and Effect Sizes for Meals In Our Household subscales, the Perceived Stress Scale-Short Form (PSS-4) and caregiver reported mealtime stress for ASC, ADHD, ASC+ADHD and NT children.*

| Measures | ASC | |  | ADHD | |  | ASC+ ADHD | |  | NT | | Group | |  | Age | |
| --- | --- | --- | --- | --- | --- | --- | --- | --- | --- | --- | --- | --- | --- | --- | --- | --- |
|  | *n* | _Adj_*M*  (*SE*) |  | *n* | _Adj_*M*  (*SE*) |  | *n* | _Adj_*M*  (*SE*) |  | *n* | _Adj_*M*  (*SE*) | *F* (DoF) | *ηp²* |  | *F* (DoF) | *ηp²* |
| **MIOH subscale** |  |  |  |  |  |  |  |  |  |  |  |  |  |  |  |  |
| Problematic Child Mealtime Behaviours (*N*=351) | 80 | 27.9 (1.15) |  | 88 | 32.8 (1.09) |  | 65 | 34.3 (1.28) |  | 118 | 15.1 (0.95) | 70.935** (3,346) | .381 |  | 46.023** (1,346) | .117 |
| Parental Concern about Child’s Diet (*N*=344) | 79 | 19.3 (1.44) |  | 84 | 17.4 (1.40) |  | 64 | 19.6 (1.61) |  | 117 | 9.8 (1.19) | 12.602** (3,339) | .100 |  | 0.164 (1,339) |  |
| Structure of Family Meals (*N*=349) | 79 | 24.2 (0.60) |  | 88 | 26.1 (0.57) |  | 64 | 23.2 (0.67) |  | 118 | 28.5 (0.49) | 17.329** (3,344) | .131 |  | 0.699 (1,344) |  |
| Spousal Stress (*N*=313) | 73 | 9.9 (0.48) |  | 76 | 12.1 (0.47) |  | 54 | 12.4 (0.56) |  | 110 | 7.1 (0.40) | 29.526** (3,308) | .223 |  | 11.448** (1,308) | .036 |
| **PSS-4** (*N*=349) | 80 | 2.1 (0.08) |  | 88 | 2.2 (0.07) |  | 65 | 2.1 (0.09) |  | 116 | 1.6 (0.06) | 15.685** (3,344) | .120 |  | 0.746 (1,344) |  |
| **Additional item** |  |  |  |  |  |  |  |  |  |  |  |  |  |  |  |  |
| Caregiver reported mealtime stress (*N*=351) | 80 | 2.2 (0.10) |  | 88 | 2.5 (0.10) |  | 65 | 2.5 (0.11) |  | 118 | 1.6 (0.08) | 23.141** (3,346) | .167 |  | 12.581** (1,346) | .035 |

Notes

*N/n* varies due to missing data. i.e., participants with missing data points in a subscale were excluded from analysis for that subscale.

*significant at *p*<.05; **significant at *p*<.001.

ANCOVA calculated for significant ANOVA subscales only.

*M* = mean; _Adj_*M* = estimated marginal mean, *SE* = standard error; *ηp²* = partial eta squared (reported for significant results only); *DoF =* degrees of freedom.
